# A proteomic analysis of LRRK2 binding partners reveals interactions with multiple signaling components of the WNT/PCP pathway

**DOI:** 10.1186/s13024-017-0193-9

**Published:** 2017-07-11

**Authors:** Alena Salašová, Chika Yokota, David Potěšil, Zbyněk Zdráhal, Vítězslav Bryja, Ernest Arenas

**Affiliations:** 1 Laboratory of Molecular Neurobiology, Department of Medical Biochemistry and Biophysics, Karolinska Institutet, 17177 Stockholm, Sweden; 2 Current address: Science for Life Laboratory, Department of Biophysics and Biochemistry, Stockholm University, 171 65 Stockholm, Sweden; 3 Central European Institute for Technology, Masaryk University, Kamenice 5, 625 00 Brno, Czech Republic; 4 Department of Experimental Biology, Faculty of Science, Masaryk University, 625 00 Brno, Czech Republic

**Keywords:** WNT/planar cell polarity, PRICKLE1, CELSR1, DVL, Parkinson’s disease, Dopaminergic neurons, *Substantia nigra*, Immunoprecipitation, Endocytosis, Signalosomes

## Abstract

**Background:**

Autosomal-dominant mutations in the *Park8* gene encoding Leucine-rich repeat kinase 2 (LRRK2) have been identified to cause up to 40% of the genetic forms of Parkinson’s disease. However, the function and molecular pathways regulated by LRRK2 are largely unknown. It has been shown that LRRK2 serves as a scaffold during activation of WNT/β-catenin signaling via its interaction with the β-catenin destruction complex, DVL1-3 and LRP6. In this study, we examine whether LRRK2 also interacts with signaling components of the WNT/Planar Cell Polarity (WNT/PCP) pathway, which controls the maturation of *substantia nigra* dopaminergic neurons, the main cell type lost in Parkinson’s disease patients.

**Methods:**

Co-immunoprecipitation and tandem mass spectrometry was performed in a mouse *substantia nigra* cell line (SN4741) and human HEK293T cell line in order to identify novel LRRK2 binding partners. Inhibition of the WNT/β-catenin reporter, TOPFlash, was used as a read-out of WNT/PCP pathway activation. The capacity of LRRK2 to regulate WNT/PCP signaling in vivo was tested in *Xenopus laevis*’ early development.

**Results:**

Our proteomic analysis identified that LRRK2 interacts with proteins involved in WNT/PCP signaling such as the PDZ domain-containing protein GIPC1 and Integrin-linked kinase (ILK) in dopaminergic cells in vitro and in the mouse ventral midbrain in vivo. Moreover, co-immunoprecipitation analysis revealed that LRRK2 binds to two core components of the WNT/PCP signaling pathway, PRICKLE1 and CELSR1, as well as to FLOTILLIN-2 and CULLIN-3, which regulate WNT secretion and inhibit WNT/β-catenin signaling, respectively. We also found that PRICKLE1 and LRRK2 localize in signalosomes and act as dual regulators of WNT/PCP and β-catenin signaling. Accordingly, analysis of the function of LRRK2 in vivo*,* in *X. laevis* revelaed that LRKK2 not only inhibits WNT/β-catenin pathway, but induces a classical WNT/PCP phenotype in vivo.

**Conclusions:**

Our study shows for the first time that LRRK2 activates the WNT/PCP signaling pathway through its interaction to multiple WNT/PCP components. We suggest that LRRK2 regulates the balance between WNT/β-catenin and WNT/PCP signaling, depending on the binding partners. Since this balance is crucial for homeostasis of midbrain dopaminergic neurons, we hypothesize that its alteration may contribute to the pathophysiology of Parkinson’s disease.

**Electronic supplementary material:**

The online version of this article (doi:10.1186/s13024-017-0193-9) contains supplementary material, which is available to authorized users.

## Background

Parkinson’s disease (PD) is one of the most common neurodegenerative disorders. Clinically, it is characterized by a triad of classical symptoms: resting tremor, rigidity or hypokinesia. At pathological level, the hallmarks of PD include a predominant loss of midbrain dopaminergic (mDA) neurons in the *substantia nigra pars compacta* (SNpc) and the presence of Lewy bodies containing aggregated α-SYNUCLEIN filaments, and/or TAU hyperphosphorylation [1, 2]. Current treatments for PD are symptomatic and do not affect the progressive nature of the degenerative process. Efforts aiming at developing more effective therapies capable of stopping or delaying disease progression currently involve gaining a better understanding of the function of proteins involved in PD. Ninety percent of the patients suffer from sporadic idiopathic forms of PD [3]. In last decades, several genetic forms of PD, accounting for only 10% of the cases, have been identified. Notably, several proteins that are implicated in genetic forms of PD, such as PARKIN, TAU, α-SYNUCLEIN, PINK1 and DJ-1, have been also associated with sporadic PD [4, 5].

Autosomal-dominant mutations in *Park8* gene encoding Leucine-rich repeat kinase2 (LRRK2) is one of the most prominent risk factors for sporadic PD with a mutation frequency of 2-40% in different populations [3, 6, 7]. Interestingly, these patients exhibit typical features of idiopathic, late-onset PD, indicating that even LRRK2-mediated disease requires aging [1, 5]. LRRK2 is a large, multi-domain protein composed of 2527 amino acids (289 kDa). It contains a kinase domain sequence, a Ras of complex protein domain (ROC) and the C-terminal of COR (COR) domain that are predicted to bind and hydrolyze GTP similarly to the ROCO protein family [8]. These three domains are considered the catalytic core of LRRK2. Additionally, LRRK2 has Ankyrin repeats, Leucine-rich repeats (LRR) and a WD40 domain that predominantly serve as binding sites for protein-protein interactions and structural scaffolds for different signaling processes, which is another important function of LRRK2. However, the precise function of LRRK2 in cell signaling remains to be defined.

WNTs (from *Win* *gless/* *Int* *egration*) are a large family of 19 secreted lipid-modified glycoproteins that serve multiple functions in development, tissue homeostasis, regeneration and disease [9]. In mammalian nervous system, WNT morphogens play a crucial role in formation and modulation of neuronal circuits [10]. Three main WNT signaling pathways have been described: WNT/β-catenin, WNT/Planar Cell Polarity (PCP) and WNT/Calcium (Ca^2+^), the two latter also referred to as non-canonical or WNT/β-catenin-independent signaling pathways. Upregulation of WNT/PCP signaling usually inhibits the WNT/β-catenin signaling and vice versa [11, 12]. We previously found that proper levels of WNT/β-catenin and WNT/PCP signaling are required for correct mDA neuron development and function [13–16].

A few studies have provided evidence that WNT/β-catenin signaling components interact with “PD proteins”. We previously found that PARKIN, an ubiquitin E3 ligase forming a complex with PINK and DJ-1 [17], interacts with β-CATENIN and regulates its degradation [18]. More recently, LRRK2 was found to interact with two key components of the WNT/β-catenin pathway, DVL1-3 [19] and LRP6 [20], bringing them together to the plasma membrane and leading to activation of this pathway.

In this study, we performed a proteomic analysis of LRRK2 interactions in the mouse *substantia nigra* cell line SN4741 and human HEK293T cells. Our findings show for the first time that there is a clear crosstalk between LRRK2 and WNT/PCP signaling. By using immunoprecipitation-coupled tandem mass spectrometry (MS/MS), we identified a number of LRRK2 binding partners involved in WNT/PCP signaling pathway, such as the PDZ-domain-containing protein GIPC1 [21] and Integrin-linked kinase (ILK) [22, 23]. We also found that LRRK2 interacts with PRICKLE1 and CELSR1, two core components of WNT/PCP pathway [24–27]. Finally, we demonstrate that LRRK2 alone or together with PRICKLE1 and DVL2 can activate the WNT/PCP signaling and suppress the expression of WNT/β-catenin dependent genes both in vitro and in vivo, during *X. laevis* development. Our data thus extends the spectra of signaling pathways interacting with LRRK2 to include WNT/PCP, a fundamental signaling pathway for mDA neuron maturation [14]. We hypothesize that adult onset of PD may involve a deregulation of WNT/PCP signaling via its interactions with LRRK2. Our data provide new insights into LRRK2 function and may contribute to gain a better understanding of PD.

## Methods

### Cell culture and transient transfection

Human embryonic kidneys 293T cells (HEK293T; ATCC) were grown in DMEM containing 10% FBS, 2 mM L-glutamine, 50 U/ml penicillin, and 50 U/ml streptomycin (all from Gibco Inv.). 24 h prior transfection HEK293T were seeded on 10 cm dishes at 40% confluence in complete medium (co-IP). For IF and TOPFlash analysis, HEK293T cells were seeded into 24-well plate with a density of 40,000 cells/well with or without 13 mm glass coverslips coated with 0.1% gelatin. For transfection, the medium was switched for DMEM only. OptiMem (Gibco Inv.) was mixed with 5 μg (2.5 μg: 2.5 μg) of DNA and 12.5 μl of Lipofectamine 2000 (Gibco Inv.) for each condition. For 24-well plates we used 10× fewer reagents. The transfection mixture was applied to the cells and incubated for 5 h. Afterwards, the medium was exchanged for complete medium. A mouse clonal *substantia nigra* dopaminergic neuron cell line SN4741 (a gift from Jong W. Lee [28]) was cultivated in similar conditions in medium containing 0.6% glucose. For co-IP experiments, SN4741 were seeded in 90% confluency 24 h prior harvest. For IF, SN4741 were seeded into 24-well plate on 13 mm glass coverslips coated with 0.1% gelatin with density of 20,000 cells/well. Cells were trypsinized with 0.05% Trypsin-EDTA (Gibco Inv.) and passaged every 3rd day in the culture.

### Plasmids

The following plasmids were used for transfections: pDEST-Prickle1-ECFP, pCMV3Tag-9-Celsr1-3xMyc, pEGFPN1-Celsr1 and pEGFPN1-Celsr1-CRASH (a gift from Elaine Fuchs), pcDNA3.1 backbone, pCMVTag-3B and pDEST51 backbones (a gift from Dr. Mark Cookson), pcDNA3.1-3xFlag-Cullin3 (a gift from Feng Shao), pCAFlag-Shroom3 (a gift from Masatoshi Takeichi), Flotillin1-GFP and Flotillin2-GFP (a gift from Ben Nichols), pcDNA3-flag-Cullin1 (a gift from Tadashi Yamamoto), pcDNA3-Vangl2-HA (a gift from Thomas Ringstedt), pcDNA3-Ror2-Flag (a gift from Sigmar Stricker), pcDNA3-Flag-JIP3 and pcDNA3-Flag-JIP4 (a gift from Roger Davis), pEGFP-C1-Rab5a (a gift from Philip D. Stahl), DsRed-Rab7-WT (from Addgene, #12661), GFP-Rab11-WT (from Addgene, #12674), pLAMP1-mCherry (from Addgene, #45147), pcDNA3.1-HA-mDvl2 wt (a gift from Mariann Bienz), pEGFP-C1 DVL2 (a gift from Bob Lefkowitz). Lrrk2 plasmids were a gift from Dr. Mark Cookson and were bought from Addgene: pCMVTag-3B-Lrrk2-WT-2xMyc (Addgene #25361), pCMVTag-3B-Lrrk2-WD40-2xMyc (Addgene #25073), pCMVTag-3B-Lrrk2-ΔHeat-2xMyc (Addgene #25068), pCMVTag-3B-Lrrk2-RCKW-2xMyc (Addgene #25064), pCMVTag-3B-Lrrk2-RCK-2xMyc (Addgene #25065), pCMVTag-3B-Lrrk2-COR-2xMyc (Addgene #25069), pCMVTag-3B-Lrrk2-Kinase-2xMyc (Addgene #25071), pCMVTag-3B-Lrrk2-Lrrs-2xMyc (Addgene #25072), pDEST51-Lrrk2-WT(V5) (Addgene #25080), pDEST51-Lrrk2-Y1699C(V5) (Addgene #25084), pDEST51-Lrrk2-R1441C(V5) (Addgene #25081), pDEST51-Lrrk2-G2019S(V5) (Addgene #29401). We used pCS2-ca-b-catenin, pCS2-14XTOPFlash, Super8X TOPFlash and pRLtkLuc vectors for TOPFlash assay (Promega). Plasmid lentiCRISPRv2, a gift from Feng Zhang (Addgene, #52961), was used to produce CRISPR/Cas9 cell lines.

### Lentivirus and CRISPR/Cas9 work

Sequence 5′-CGCCTGTCAGGGCTGCGAAG-3′ targeting exon 1 of Park8 gene was used for CRIPSR/Cas9 driven Lrrk2 knock-down; sequence 5′-GAAGTTCGAGGGCGACACCC-3′ targeting an irrelevant gene, EGFP, was used for the control cell line [29]. The two targetting sequences were cloned into sgRNA lentiviral vector lentiCRISPR-V2 (a gift from Feng Zhang; Addgene, #52961) using Golden-gate sgRNA cloning protocol as decribed by the Zhang lab [30] and sequenced with U6 sequencing primer ACTATCATATGCTTACCGTAAC. All plasmids were purified prior the transfection with an endotoxin-free Zymopure Maxiprep kit (Zymo research, #D4202). We used 3rd generation of replication deficitent lentiviruses (packing vectors pMD2.G and psPAX/2) for the viral production following the guidlines of local ethical committee (Arbetsmiljöverket, permit Dnr 2-5164/2016). For the virus production, we first transiently overexpressed our cloned transfer vectors lentiCRISPR-V2 (10 μg), and packaging vectors pMD2.G (5 μg) and psPAX/2 (7.5 μg) in 80% confuent HEK293FT cells grown on 10 cm dishes using Lipofectamine 2000 (Invitrogene) mixed with OptiMEM (Invitrogene). The transfection mixture was incubated with cells in serum and antibiotics-free medium for 5 h. Afterwards, the medium was switched for the complete DMEM enriched with 10%FBS and 1% Pen/Strep. Medium with viral particles was collected twice 48 h and 72 h post transfection, and pooled together. The medium was centrifuged (200 g, 5 min, room temperature) to clear from the cell debris. The supernatants were subsequently filtered through 0.45 μm low protein binding membrane filters (Millipore). The viral supernatants were centrifuged at 60.000 g for 2 h at +4 °C. The viral pelets were resuspended in 200 μl of PBS after overnight incubation at +4 °C. For the CRISPR/Cas9 knock-down, the SN4741 cells were seeded at 40% confluency on 6-wells plate 24 h prior transduction. The next day, 1 ml of fresh complete medium was pre-mixed with 8 μg/ml of polybrene (Millipore) for better transduction efficacy and added to the cells together with 20 μl of lentiviruses per well, overnight. We included a control sample which was not infected. We exchanged the medium for a fresh one the next day. We started the puromycin selection (4 μg/ml; ThemoFisher Scientific) 48 h post transduction. The cells were held under selection for 10 days. Cells in the uninfected sample all died after puromycin treatment within 48 h compared to the positive clones. Single clones were picked using a single cell serial dilution and tested for LRRK2 knock-down using specific LRRK2 antibodies (Fig. 2, Additional file 1: Figure S1A-B). The precise allelic disruption was determiend using T7E1 and DNA sequencing. These cell lines are available upon a request.

### Nested-PCR, T7E1 assay and DNA sequencing

We used a nested-PCR followed by T7E1 and DNA sequencing (Eurofins) to characterize mutations in our CRISPR/Cas9 cell lines. Genomic DNA of the CRISPR/Cas9 derived cell lines was isolated according the manufacturer’s instructions using NucleoSpin Tissue (Macherey-Nagel, Germany). Isolated genomic DNA was subjected to the nested-PCR with the Q5 high-fidelity polymerase (New England Biolabs). The following primers were used sequentially: the first PCR primers 5′-GAAACCGCTTTCCTGAAAGG-3′ and 5′-GGTGCCCAAGATTAAGACTC-3′, and the second PCR primers 5′-GCCCCTTTGCTATTCTTAGT-3′ and 5′-AAAGTTTGCAGAGGAGGGAG-3′. 200 ng of genomic DNA and 0.2 μM of the primers were used for the first PCR in a final volume of 50 μl, with the PCR set up as: 95 °C for 15 s, 60 °C for 15 s, 72 °C for 30s X 20 cycles. 1 μl of each PCR product from the first PCR was subjected to the second PCR in a final volume of 50 μl with 0.2 μM of primers. The conditions of the second PCR were: 95 °C for 15 s, 60 °C for 15 s, 72 °C for 30s X 35 cycles. 418 bp product of each PCR was purified with QIAquick gel extraction kit (Qiagen). The sequences of the products were determined with the second PCR primers and DNA sequencing (Eurofins). The T7E1 assay was used to distinguish whether our clonal LRRK2 cell line carries mono- or biallelic mutations. 1 μg of gel-purified PCR products were subjected to the T7E1 assay. 1XNEB buffer 2.1 and 1 μg of PCR products in a final volume of 50 μl were heated at 95 °C for 5 min and gradually cooled down to room temperature. 25 μl of the products were digested with 5 U of T7E1 (New England Biolabs) and incubated at 37 °C for 45 min. The rest of the products (25 μl) were incubated without T7E1, and used as a control.

### Protein extraction, co-immunoprecipitation

For co-IP of overexpressed proteins, proteins were extracted from cells 24-48 h post transfection. For co-IP of endogenous LRRK2, SN4741 cells were lyzed in 90% of confluency 24 h after seeding. Mouse VM tissue was first frozen to −80 °C and lysed once needed. Cells were washed twice in ice-cold PBS, and lyzed in 1-2 ml of freshly prepared lysis buffer (50 mM Tris pH 7.6, 150 mM NaCl, 1 mM EDTA, 0.5% NP40, 0.1 mM DTT and proteases inhibitors cocktail from Roche) on ice for 15 min. The lysis was followed by centrifugation of the lysates at 18000 g, +4 °C for 20 min to separate insoluble particles and cellular debris. To eliminate unspecific interactions, lysates were pre-cleared by incubating the lysates with DynaBeads (Invitrogen) for 45 min. To pull down the binding partners, 800 μl of lysates were incubated with 1 μg of following antibodies: goat GFP-FITC (Abcam), rabbit C-MYC (Sigma), mouse V5 (Invitrogene), rabbit FLAG (Sigma) and mouse LRRK2 (Covance), while rotating at +4 °C for 3 h. Afterwards, the protein complexes were incubated with DynaBeads for 12-14 h. The beads were washed 5 times using lysis buffer without DTT and proteases inhibitors. To elute the complexes from the beads, the beads were mixed with 1× Laemmli buffer and denaturized at 95 °C for 5 min. The beads were subsequently removed and samples loaded directly into 8-10% SDS-PAGE gel.

### Immunoblotting

Western blotting was used to analyze the LRRK2 interactions. The samples were subjected to polyacrylamide SDS-PAGE gel electrophoresis. Proteins were transferred onto PVDF membrane (GE-Healthcare, Germany, US) and immunoblotted with primary antibodies at 4 °C overnight (Additional file 2: Table S1). After washing, membranes were incubated with appropriate HRP-conjugated secondary antibodies (1:5000, Sigma-Aldrich) at room temperature for 1 h. Signals were detected with the Amersham ECL Prime system (GE-Healthcare) using a charge-coupled device camera (Bio-Rad, US). Not-saturated immunoblots were analyzed by ImageJ.

### Immunofluorescence and confocal microscopy

HEK293T were transfected and fixed 24-30 h post transfection in 4% PFA at RT for 20 min. SN4741 cells were fixed in the same way, 24 h after seeding. Subsequently, cells were washed in PBS-Tween20 (PBT, 0.5%) 3 times for 5 min each, blocked in PBTA with 5% of donkey serum in room temperature for 45 min, and incubated in +4 °C overnight with primary antibodies (Additional file 2: Table S1). Next day, cells were washed in PBT 4 times for 5 min each and incubated with particular secondary Alexa Fluor antibodies (Additional file 2: Table S1). Samples were washed 3 times in PBT for 5-10 min in room temperature and incubated with Dapi (1:5000, Invitrogene) for other 10 min. Coverslips were mounted with Fluorescent mounting medium (S3023, Dako). Confocal imaging was performed with Zeiss LSM700, 63× oil objective.

### Dual Luciferase reporter assay (TOPFlash assay)

HEK293T were transfected with 20 ng of pRLtkLuc as a control for overall translation machinery, 200 ng of TCF/LEF reporter Super8X TOPFlash, and with 200 ng of each plasmid of interest (empty backbones, V5-Lrrk2 or Myc-Lrrk2, Prickle1-ECFP and Dvl2-EGFP) per well. Altogether, we used from 3 (420 ng) to 5 plasmids (820 ng DNA) per well. Cells were lysed 27 h post transfection. The basal activity of WNT/b-catenin signaling corresponds to the negative control where only empty backbones (pCMV-back, pDEST51-back and pcDNA3) were transfected. Samples were analyzed with dual luciferase reporter assay kit (Promega). The samples were measured either with Victor (PerkinElmer) (Fig. 5b-d) and/or with more sensitive Omega Fluor plate-reader (Fig. 5d-e). Subsequently, all data were first normalized to Renilla signal. Values were normalized to overexpression of proteins that did not differ from our backbone control, Prickle1 in Fig. 5b and Lrrk2-WT in Fig. 5c. Results in Fig. 5d and e are presented as RAW values (referred to as RLU) normalized to Renilla. For the *X. laevis* microinjections, 14XTOPFlash reporter plasmid was created by inserting 14 tandem repeats of TCF binding sites into pGL4.26 (luc2/minP/Hygro) (Promega). 200 pg of 14XTOPFlash and 25 pg of renilla (pRL Renilla; Promega) DNA were co-injected with mRNAs into two animal cells at 4 to 8cell-stage embryos, and firefly and renilla luciferase were measured at st10.25 *Xenopus* embryos with Dual-Glo Luciferase Assay System (Promega). Triplicate samples (three embryos/each) were used for an individual condition, and three independent experiments were examined. The graphs and statistical analysis were prepared in GraphPad Prism6 using one way ANOVA test combined with Holm-Sidak multi-comparison test or Mann Whitney T-test for the HEK293T samples, and paired student T-test for the frog samples.

### Collection of mouse tissue, ethical permits

Ventral midbrains of C57/BL6 wild type mice were collected from E18.5 embryos. Mice were housed, bred and treated according to the guidelines of the European Communities Council (directive 86/609/EEC) and the local ethics committees (Stockholm’s Norra Djurförsöketiska Nämnd N158/15).

### Preparation of *Xenopus laevis* embryos and microinjections

We followed institutional guidelines for animal care and research protocols, and approved by the Etiska Nämnden on animal use (ethical permit N241/14). *Xenopus laevis* eggs were obtained by injecting frogs with 700 units of human chorionic gonadotropin (Pregnyl®, Merck Sharp & Dohme). The embryos were fertilized using a sperm suspension and were dejellied with 1% thioglycolic acid at two-cell stage, and cultured in 0.2× Marc’s Modified Ringer’s solution (MMR) at 18-21 °C. Staging was according to Nieuwkoop and Faber [31]. Microinjections were performed in 4% Ficoll/0.3× MMR. The maximum injection volume was 20nl per embryo. The embryos were then cultured in 0.2X MMR until either stage 10.25 (for TOPFlash assay) or stage 32 (for the PCP phenotypes). The mMessage mMachine® sp6 Kit (Ambion) was used to synthesize in vitro capped mRNA. Lrrk2 construct used for the experiments was pCS2-5Xmyc-Lrrk2 [32]. pCS2-super was generated by inserting an oligonucleotide fragment containing a polylinker sequence (EcoRI, PacI, SbfI, XmaI, XhoI, AscI, XbaI) into the EcoRI/XbaI sites of pCS2. To generate pCS2-beta-galactosidase, beta-galactosidase with nuclear localization signal was obtained by RT-PCR from pBSApBpACAGftILn [33], and subcloned into the PacI/AscI sites of pCS2-super.

### Analysis of overexpressed LRRK2 interactors in HEK293T cells by LC-MS/MS

HEK293T cells were transiently transfected with human 2xmyc-Lrrk2-WT plasmid, and lysed in NP40-buffer 24 h post transfection. The eluted samples were subsequently loaded on polyacrylamide gel, followed by silver staining and in-gel tryptic digestion.

#### In-gel digestion of silver-stained gel bands

Each gel lane was divided into 20 bands, which were de-stained in 50 mM ammonium bicarbonate and 50% acetonitrile. Tryptic digestion was performed by a liquid-handling robot (MultiProbe II, Perkin Elmer), including protein reduction in 10 mM DTT and alkylation in 55 mM iodacetamide. Gel pieces were dehydrated in 100% acetonitrile, trypsin was added to a final concentration of 13 ng/μl, and the pieces were digested over night at 37 °C. Extracted peptides from consecutive bands were pooled according to their protein levels, resulting in two pools for each lane.

#### Liquid chromatography tandem mass spectrometry

Nano-LC-MS/MS analyses were performed using an Easy-nLC system (Thermo Scientific) directly coupled to an Orbitrap Q Exactive mass spectrometer (Thermo Scientific). Peptides were separated in a 10-cm fused SilicaTip column (New Objective, Inc.) that was in-house packed with 3-μm C18-AQ ReproSil-Pur (Dr. Maisch GmbH) using a linear gradient from 3 − 48% acetonitrile in 89 min at a flow rate of 300 nl/min. The MS acquisition method was comprised of one survey full scan ranging from m/z 300 to m/z 1650 acquired at a resolution of *R* = 70′000 at m/z 400, followed by up to ten data-dependent HCD MS2 scans from the top ten precursor ions with a charge state ≥2 and at *R* = 17,500.

### Analysis of endogenous interactors of LRRK2 in SN4741 cells by LC-MS/MS

SN4741 cells were lysed either in NP40-buffer, followed by SDS-PAGE and Coomassie staining and processed in gel; or lysed in a buffer containing 0.1% sodium deoxycholate and processed for MS/MS analysis via tryptic digestion directly on beads.

#### On-beads and in-gel protein digestion

Beads with extracted proteins were washed 3× by 50 mM ammonium bicarbonate buffer and subjected to digestion by trypsin (2 h, 37 °C; sequencing grade, Promega). Tryptic peptides were extracted into LC-MS vials by 2.5% formic acid (FA) in 50% ACN with addition of polyethylene glycol (20,000; final concentration 0.001%) and concentrated in a SpeedVac concentrator (Thermo Fisher Scientific). 1D gel lines for sample and negative control were divided into 10 gel areas separating gel areas with high and low abundant proteins. Individual gel areas were excised manually and after destaining and washing procedures each band was incubated with 125 ng trypsin (sequencing grade; Promega). The digestion was performed for 2 h at 40 °C on a Thermomixer (750 rpm; Eppendorf). Tryptic peptides were extracted as mentioned above.

#### LC-MS/MS analysis of peptides

LC-MS/MS analyses of peptide mixture were done using RSLCnano system connected to Orbitrap Elite hybrid mass spectrometer (Thermo Fisher Scientific). Prior to LC separation, tryptic digests were desalted using trapping column (100 μm × 30 mm, 3.5-μm X-Bridge BEH 130 C18 sorbent, Waters) and separated on Acclaim Pepmap100 C18 column (2 μm particles, 75 μm × 500 mm; Thermo Fisher Scientific) by one or two hour gradient program (mobile phase A: 0.1% FA in water; mobile phase B: 0.1% FA in acetonitrile). The analytical column outlet was directly connected to the Nanospray Flex Ion Source. Up to top 10 precursors from the survey scan (350-2000 m/z, resolution 60,000) was selected for HCD fragmentation (target 50,000 charges; resolution 15,000, isolation window 2 m/z) with enabled dynamic exclusion (up to 45 s). Two independent LC-MS/MS analyses were done for on-beads digests. The analysis of the mass spectrometric RAW data files was carried out using the Proteome Discoverer software (Thermo Fisher Scientific; version 1.4) with in-house Mascot (Matrixscience; version 2.3.1) and Sequest search engines utilization. MS/MS ion searches were done against UniProtKB protein database for mouse (downloaded from ftp://ftp.uniprot.org/pub/databases/uniprot/current_release/; version 20141001; 85,893 protein sequences) with additional sequences from cRAP database (downloaded from http://www.thegpm.org/crap/). Mass tolerance for peptides and MS/MS fragments were 10 ppm and 0.05 Da, respectively. Oxidation of methionine and deamidation (N, Q) as optional modification and two enzyme miss cleavages were set for all searches. Propionamidation of C as optional modification was set for MS database searches after in-gel digestion. Percolator was used for post-processing of Mascot search results. Peptides with false discovery rate (FDR; q-value) < 1%, rank 1 and with at least 6 amino acids were considered. Proteins matching the same set of peptides were reported as protein groups. Protein groups/proteins were reported only if they had at least one unique peptide. Label-free quantification using protein area calculation in Proteome Discoverer was used (“top 3 protein quantification”). Two analyses of on-beads digests and analyses for all gel area within single gel line were searched as one dataset.

### Database search

Tandem mass spectra were extracted using Raw2MGF (KI in-house software), and the resulting mascot generic files from each lane were searched against the mouse SwissProt protein database using the Mascot Daemon 2.3.02 search engine (Matrix Science Ltd.), which was set to search the SwissProt protein database (selected for Homo s. sapiens, version 2012.03) using trypsin and two missed cleavage sites. Peptide mass tolerance was set to 10 ppm and 0.05 Da for the fragment ions. Carbamidomethylation of cysteine was specified as a fixed modification, whereas oxidation of methionine and deamidation of asparagine and glutamine were defined as variable modifications. The lists of identified proteins were exported from the .dat files using the following criteria: significance threshold of 0.05, MudPit protein scoring, required red bold, and including same-set proteins. Mascot score threshold was set to 50.

### MS/MS data mining

We performed a literature study on identified proteins using UniProt [34], PubMed and Web of Science databases. Overexpression studies were performed in HEK293T, therefore the protein IDs had to be converted into mouse Ids, and not all the hits were present in the mouse genome. The conversion of the protein ID was done by **bio**logical **D**ata**B**ase **net**work (bioDBnet).

## Results

### Unbiased identification of WNT/PCP proteins as candidate LRRK2 binding partners

**Fig. 1:**
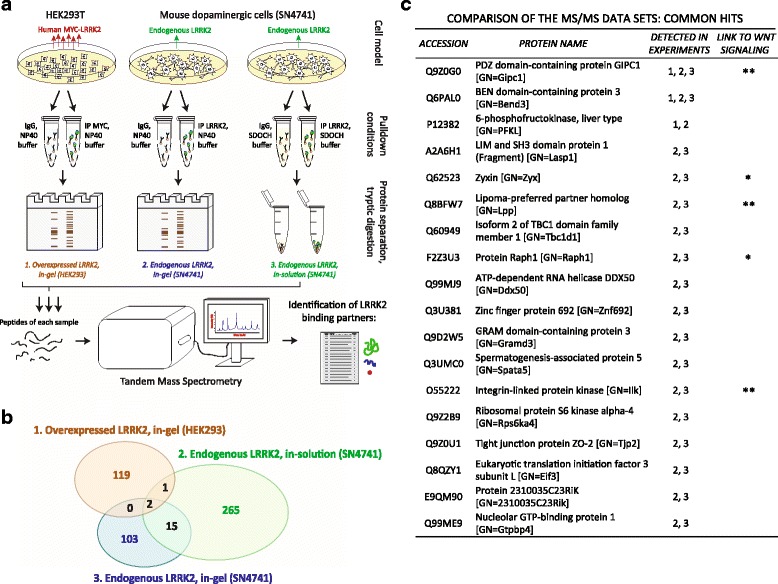
Large-scale immunoprecipitation-coupled MS/MS screening of LRRK2 binding partners revealed proteins belonging to WNT/PCP signaling pathway. **a** Scheme of the experimental workflow. **b** Venn diagram showing results from 3 different MS/MS data sets. **c** A list of identified proteins which were detected at least in 2 experiments. Proteins that have been linked to WNT signaling are marked with a star, stronger evidence is denoted by with two stars

Proteomic approaches such as tandem mass spectrometry (MS/MS) has become a powerful tool to discover unknown binding partners of any protein of interest. Nevertheless, the stoichiometry of protein-protein interactions and their preservation in a test tube is highly dependent on the cell type and sample processing [35–37]. To capture the diversity of candidate LRRK2 interactions, we used three different conditions: (1) NP40 buffer and in-gel tryptic digestion of human HEK293T; (2) NP40 buffer and in-gel tryptic digestion of a mouse *substantia nigra* cell line (SN4741) [28] expressing endogenous LRRK2; (3) *S*odium deoxycholate buffer (SDOCH) and digestion on beads using SN4741 cells (Fig. 1a). Only proteins absent in the IgG immunoprecipitation control, but present in the LRRK2 pull-down, were considered as candidate LRRK2 binding partners. LRRK2 was never present in IgG controls (Additional file 3: Figure S2A, Additional file 4: Table S2 and Additional file 5: Table S3), but was one of the most abundant hits in our MS/MS analysis after pull-down with antibodies against C-MYC (for LRRK2 overexpression in human cells) or endogenous LRRK2 in mouse cells, confirming the good quality of our IP protocols. This strategy allowed us to identify about 500 proteins: 122 proteins interacting with *overexpressed LRRK2 in HEK293T cells (in gel digestion)*, 120 proteins with *endogenous LRRK2 in SN4741 cells (in gel digestion)*, and 283 proteins with *endogenous LRRK2 in SN4741 cells (in solution digestion).* The full list of proteins is provided in Additional file 4: Table S2 and Additional file 5: Table S3. Comparison of the 3 data sets (Fig. 1b) revealed only 2 proteins in common: BEN domain-containing protein 3 (BEND3), a repressor of transcription, and GIPC1, a PDZ domain containing protein (Fig. 1c). Notably, 15 proteins were found to bind endogenous LRRK2 in SN4741 dopaminergic cells in the two conditions analyzed (Fig. 1b-c). Analysis of the identified LRRK2 binding partners revealed the presence of 5 proteins that play an important role in WNT/PCP signaling, either via endocytic trafficking of the core WNT receptors or by direct modulation of WNT/PCP activity (asterisk in Fig. 1c, Additional file 6: Table S4). In addition, each of our conditions detected proteins that had been previously reported to bind LRRK2 (Table 1), confirming thus the validity of our approach.

**Table 1:** Previously reported LRRK2 binding partners from the Overexpressed LRRK2 MS/MS data set were confirmed our “Overexpressed LRRK2 in-gel” data set, proving the quality and validity of the experiment

PROT_HIT	ACCESSION	PROTEIN NAME	REF	METHOD, MODEL
27	TBB4A_HUMAN	Tubulin beta-4A chain	[103]	MS and Co-IP of overexpressed LRRK2-WT in HEK293T
35	JIP4_HUMAN	C-Jun-amino-terminal kinase-interacting protein 4	[40]	Overexpression with LRRK2-WT in HEK293T
56	RIF1_HUMAN	Telomere-associated protein RIF1	[104]	MS and Co-IP of overexpressed G2019S in HEK293T
180	MYBB_HUMAN	Myb-related protein B	[104]	Its interactor RBBP4-6-7 binds to LRRK2. MS and Co-IP of overexpressed G2019S in HEK293T
184	H90B2_HUMAN	Putative heat shock protein HSP 90-beta 2	[105]	Co-IP of endogenous LRRK2 from forebrain and overexpressed in HEK293T
194	JIP3_HUMAN	C-Jun-amino-terminal kinase-interacting protein 3	[40]	Overexpression with LRRK2-WT in HEK293T
215	CENPF_HUMAN	Centromere protein F	[104]	MS and Co-IP of overexpressed G2019S in HEK293T

### In vitro and in vivo validation of GIPC1 and ILK, two novel WNT/PCP binding partners of LRRK2

**Fig. 2:**
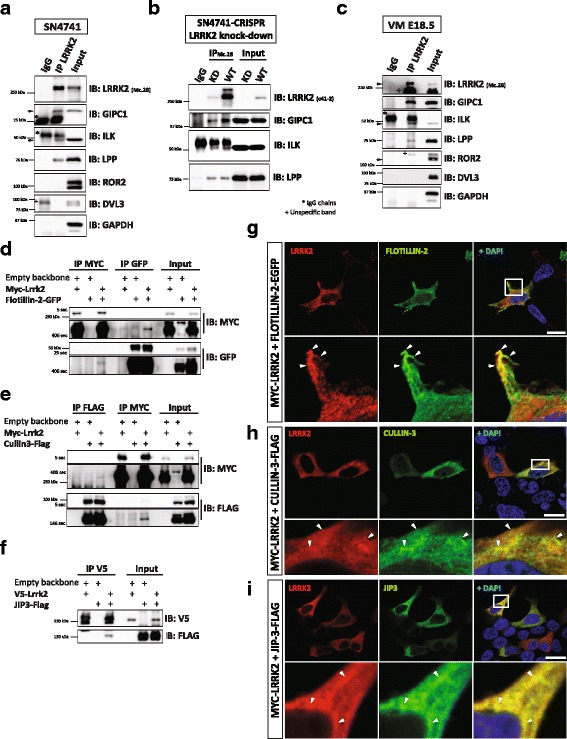
Verification of the MS results in vitro and in vivo in mouse ventral midbrain. (**a**-**c**) Western blot validation of LRRK2 binding partners using specific antibodies. ILK and GIPC1 but no other WNT/PCP signaling components interact with LRRK2 in SN4741 (**a**-**b**) and mouse ventral midbrain of E18.5 old embryos (**c**). **b** We knocked down LRRK2 using CRISPR/Cas9 methodology and generated clonal cell lines with either normal (WT) or decreased levels of LRRK2 (KD). SN4741-LRRK2-KD served as a negative control for our co-IP experiments. **d-f** Western blot analysis of co-IP of transiently overexpressed candidates shows that LRRK2 binds to FLOTILLIN-2 (**d**) and CULLIN-3 (**e**) in HEK293T. JIP3 (**f**), a previously published LRRK2 binding partner, served as positive control. **g-i** IF confirmed that LRRK2 co-localizes with FLOTILLIN-2 (**g**), CULLIN-3 (**h**), and JIP3 (**i**) in HEK293T cells. Nuclear staining by Dapi is in blue. *N* ≥ 3. Scale bars indicate 20 μm

We subsequently validated some of the candidate LRRK2 binding partners in the SN4741 *substantia nigra* cell line (Fig. 2a-b) and in embryonic day E18.5 in ventral midbrain tissue (Fig. 2c) using LRRK2 immunoprecipitation and immunoblotting. These experiments revealed that LRRK2 binds to GIPC1 and ILK in vitro (Fig. 2a) and in vivo (Fig. 2c). To further verify these results, we used three different monoclonal antibodies to compare (Additional file 1: Figure S1A-B) a control GFP SN4741 cell line to a CRISPR/Cas9 LRRK2 knock-down (KD) SN4741 cell line with monoallelic frameshift mutation (Additional file 7: Figure S3A-B). We found that the pull down of GIPC1 or ILK with LRRK2 was reduced in LRRK2 KD SN4741 cells compared to control SN4741 cells, indicating the specificity of the interaction (Fig. 2b). These experiments therefore confirmed that LRRK2 physically binds to: (1) GIPC1, an adaptor protein for G protein coupled receptors (GPCRs) that controls endocytic trafficking of the WNT/PCP receptor VANGL2 [21]; and (2) Integrin-linked kinase (ILK), an Ankyrin repeat containing serine-threonine kinase that regulates WNT/PCP [22, 23] and WNT/β-catenin [38, 39] signaling via remodeling extracellular matrix and cell-cell adhesion. Another interesting candidate from our analysis, Lipoma-preferred partner (LPP) bound to LRRK2 in SN4741 cells and in VM tissue (Fig. 2a, c) but we failed to confirm this interaction in our knock-down LRRK2 cell line (Fig. 2b). Thus our results identify two novel interactors of endogenous LRRK2 in dopaminergic cells: the WNT/PCP signaling components, GIPC1 and ILK.

### FLOTILLIN-2 and CULLIN-3 are novel WNT binding partners of LRRK2

Our in silico analysis of the identified LRRK2 interactors in HEK293T cells revealed 6 functional group of proteins (Additional file 3: Figure S2B), including a group of proteins potentially involved in WNT signaling, such as FLOTILLIN-1/2, CULLIN-1 and SHROOM-3 (Additional file 3: Figure S2C). These candidates were validated by transient overexpression and immunoprecipitation of LRRK2. We used JIP3 as positive control for LRRK2 co-IP [40]. While we did not detect any interaction of LRRK2 with CULLIN-1 or SHROOM-3, and only a weak interaction with FLOTILLIN-1 (data not shown), we confirmed that LRRK2 interacts with FLOTILLIN-2 (Fig. 2d) and JIP3 (Fig. 2f). We also found an interaction of LRRK2 with CULLIN-3 (Fig. 2e), a protein that was not identified by MS/MS, but it is known to be down-regulated by Lrrk2 knock-down [41], and to inhibit WNT/β-catenin signaling [42]. In addition, we also found that LRRK2 immunofluorescence is present in cell lamellipodia, together with FLOTILLIN-2 (Fig. 2g), or in the cytoplasm of SN4741 cells together with CULLIN-3 and JIP3 (Fig. 2h-i), reinforcing the concept that LRRK2 interacts with several proteins regulating WNT signaling in dopaminergic neurons.

### LRRK2 interacts with PRICKLE1 and CELSR1 but not with other core components of WNT/PCP pathway

**Fig. 3:**
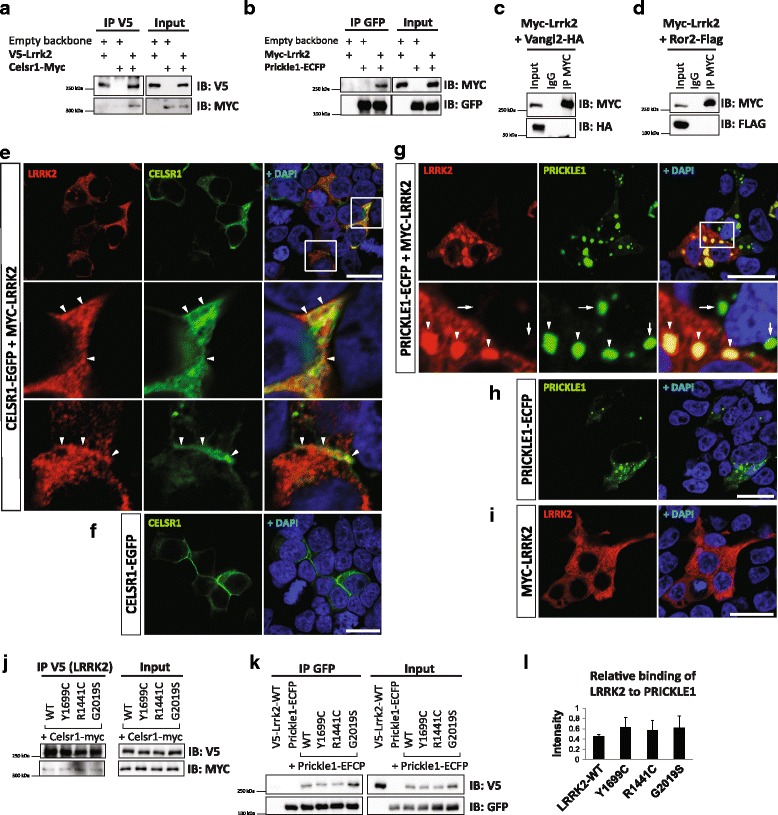
LRRK2 interacts with CELSR1 and PRICKLE1, two key components of WNT/PCP signaling. **a**-**d** Western blotting analysis of co-IP of transiently overexpressed LRRK2 with WNT/PCP signaling components in HEK293T. LRRK2 physically interacts with CELSR1 (**a**) and PRICKLE1 (**b**), but neither with VANGL2 (**c**) nor ROR2 (**d**). **e**-**i** IF of proteins overexpressed in HEK293T. Under normal conditions, LRRK2 is evenly distributed in the cytoplasm (**i**). CELSR1 alone is usually polarized in cells (**f**), whereas PRICKLE1 tends to form puncta (**h**). **e** Once co-expressed, LRRK2 partially co-localizes with CELSR1 in the cytoplasmic membrane and in cell-cell contacts (*arrowheads*). LRRK2 robustly changes its localization when it is co-expressed with PRICKLE1, and together they form puncta structures in the cytoplasm (**g**). *Arrowheads* show co-localizations, whereas *arrows* point out that there is no leakage of the fluorescent signal in PRICKLE1-positive and LRRK2-negative cells. Nuclear staining by Dapi is in *blue*. *N* ≥ 3. Scale bars show 20 μm. **j**-**l** Physical interaction between LRRK2 and CELSR1 (**j**) or PRICKLE1 (**k**-**l**) is not modified in the most common LRRK2 mutations. **l** Analysis of immunoblot band intensity shows the relative binding of LRRK2 WT and mutants to PRICKLE1. No difference between LRRK2 WT and mutants was detected. Signal was adjusted to the background and normalized to the corresponding input (*N* = 3, SD)

To further determine whether there is a possible crosstalk between LRRK2 and WNT/PCP signaling, we performed co-IP and immunoblotting experiments in HEK293T cells after overexpression of LRRK2 and some WNT/PCP signaling components, such as Ror2, Vangl2, Prickle1, and Celsr1. We found that LRRK2 specifically interacts with CELSR1 (Fig. 3a) and PRICKLE1 (Fig. 3b) but not with other key components of the WNT/PCP signaling pathway, such as VANGL2 (Fig. 3c) or ROR2 (Fig. 3d). LRRK2-CELSR1 interaction was further confirmed by using different vectors and antibodies for pull-down (Additional file 8: Figure S4A). The efficiency of the pull-down increased when more LRRK2 and less CELSR1 was transfected (Additional file 8: Figure S4B). LRRK2 immunofluorescence was found in the plasma membrane and cell-cell contacts, together with CELSR1 (Fig. 3h-i), while it was detected in cytoplasmic punctate structures together with PRICKLE1 (Fig. 3j-l), following a pattern similar to that of polymerized DVL [19, 43–46]. We also examined whether the physical interactions between LRRK2 and CELSR1 or PRICKLE1 were modified by some of the most common PD-related LRRK2 mutations, such as Y1699C, R1441C and G2019S (Fig. 3e-g). However, we did not observe any alteration in the physical interaction of these proteins, indicating that LRRK2 mutations do not affect the capacity of LRRK2 to bind core WNT/PCP proteins.

### Localization of LRRK2 into PRICKLE1-induced puncta is independent of endosomal processes and form signalosome-like structures

**Fig. 4:**
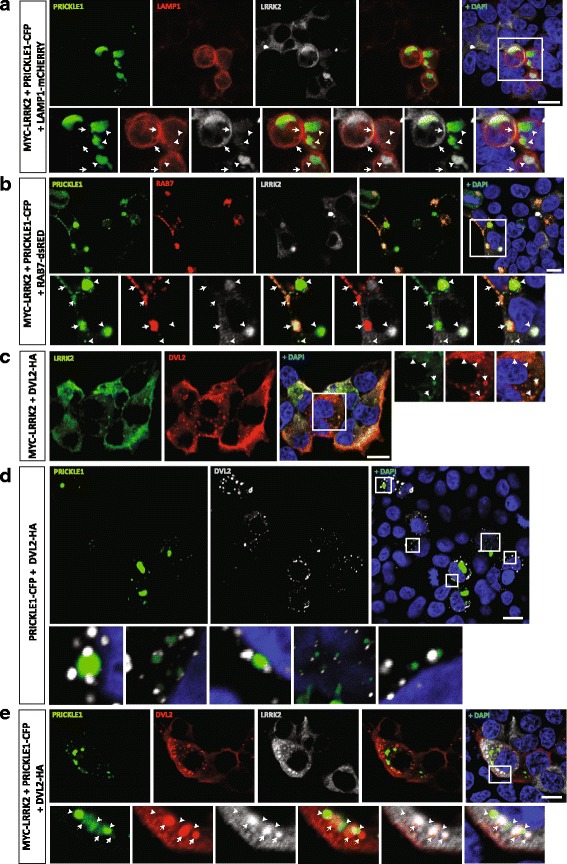
PRICKLE1-induced LRRK2 puncta are not in the endosomal compartment but rather in signalosome-like structures, together with DVL2 polymers. **a**-**e** Immunofluorescence analysis of the subcellular localization of transiently overexpressed LRRK2. **a** Areas double positive for LRRK2-PRICKLE1 are negative for the lysosomal marker LAMP1. *Arrows* point to the partial co-localization of LRRK2 with LAMP1 in the membrane. *Arrowheads* point to LAMP1, which does not localize with LRRK2-PRICKLE1 puncta. **b** A late endosomal marker, RAB7, co-localizes with PRICKLE1 (*arrowheads*), but not with LRRK2 puncta (*arrows*). **c** LRRK2 forms puncta with DVL2. **d** PRICKLE1 and DVL2 are found in close proximity, but do not co-localize. PRICKLE1 is surrounded by 1 to 3 DLV2 complexes. **e** In the presence of LRRK2, DVL2 and PRICKLE1 partially co-localize (*arrows*). The co-localization of LRRK2, DVL2 and PRICKLE1 reveals the capacity of these proteins to form signalosome-like structures. *Arrowheads* show the localization of PRICKLE1 in close proximity to DVL2 puncta and LRRK2. Nuclear staining by Dapi is in blue. *N* ≥ 3. Scale bars show 10 μm

It has been previously shown that LRRK2 interacts with several RAB family proteins and regulates the endosomal-lysosomal pathway [47–50]. We thus examined whether LRRK2-PRICKLE1 puncta are present in endosomal and/or lysosomal compartments in transiently transfected HEK293T cells. We found that LRRK2 does partially co-localize with RAB5a (marker of early endosomes) and very sporadically with RAB11 (marker of recycling endosomes), but does not co-localize with RAB7, a marker of late endosomes (Additional file 9: Figure S5A-C). We also found that PRICKLE1 as well as DVL2 strongly co-localize with RAB7 (Additional file 8: Figure S4C-D), while LRRK2-PRICKLE1 puncta neither co-localize with this late endosomal marker, nor with the lysosomal marker LAMP1, despite LRRK2 alone co-localized with LAMP1 in the cytoplasmatic membrane (Fig. 4a-b, Additional file 9: Figure S5). These results suggest that LRRK2-PRICKLE1 puncta, unlike LRRK2 or PRICKLE1 alone, are not localized in the endosomal or lysosomal compartments.

We next examined whether LRRK2-PRICKLE1 localize in DVL puncta, a WNT signaling compartment formed by DVL polymers [43–46]. First, we demonstrated that LRRK2 localizes in DVL2 puncta (Fig. 4c), as previously reported for DVL1 and DVL3 [19]. Strikingly, PRICKLE1 was found in very close proximity to DVL2, but they did not co-localize (Fig. 4d). However, in the presence of LRRK2, then PRICKLE1 and DVL2 co-localized (Fig. 4e), suggesting that LRRK2 facilitates this process. Combined, our results show that LRRK2-PRICKLE1 puncta are not in endosomes, but rather in DVL2 signalosomes, which suggests a possible regulation of WNT signaling.

### LRRK2 inhibits WNT/β-catenin signaling pathway, while PRICKLE1 turns LRRK2 from an inhibitor to an activator of WNT/β-catenin signaling

**Fig. 5:**
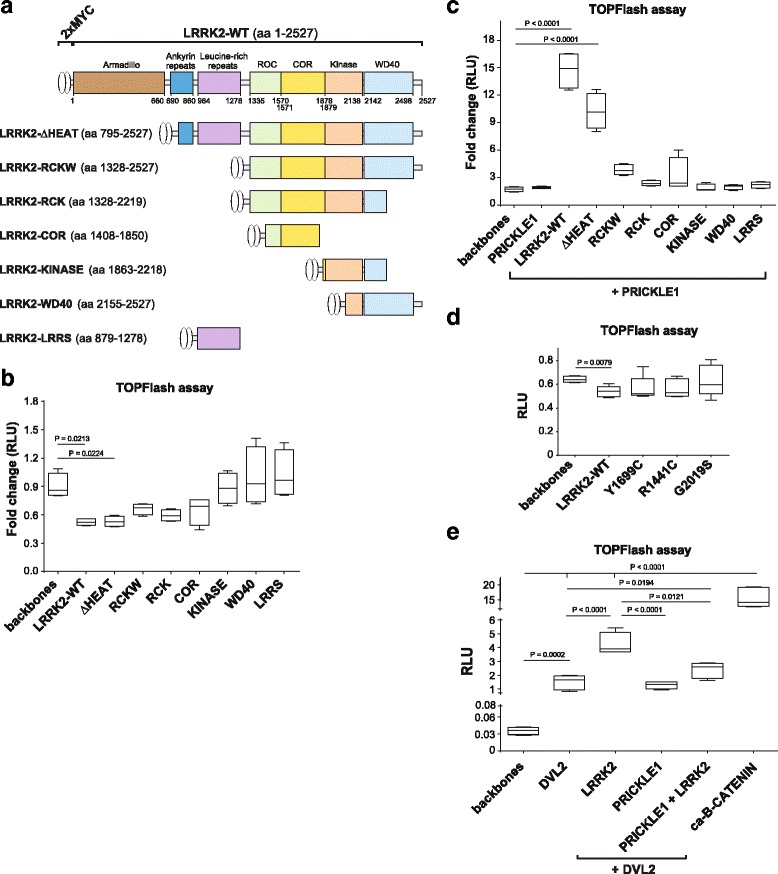
LRRK2 inhibits the activity of WNT/β-catenin pathway – an indirect read-out of WNT/PCP signaling activity. **a** Scheme of the Lrrk2 truncated vectors. **b** TOPFlash assay in HEK293T cells overexpressing LRRK2-WT or truncations. LRRK2 suppresses the basal activity of WNT/β-catenin signaling. This effect is lost with the most severe LRRK2 truncations, LRRK2-KINASE, WD40 and LRRS, that lack RocCOR domain. **c** LRRK2 together with PRICKLE1 increases the TOPFlash activity. This effect is lost in mutants that lacked the LRRs domain and the Ankyrin repeats, but is partially maintained in the mutant missing only the Armadillo domain (dHEAT). **d** LRRK2 PD mutants did not significantly inhibit TOPFlash (Mann Whitney t test). **e** DVL2 alone and together with LRRK2 strongly activates WNT/β-catenin signaling. PRICKLE1 down regulates the DVL2-dependent activation even in presence of LRRK2. ANOVA with Holm-Sidak multi-comparison test and Mann Whitney T-test (for the PD mutants) was used for statistical analysis. Data show mean (N>3) and standard deviation

There are no sensitive in vitro assays to measure the activity of WNT/PCP signaling at present. Nevertheless, it’s known that the activation of WNT/PCP signaling inhibits the WNT/β-catenin signaling [25, 42]. Here we examined whether LRRK2 and its deletion mutants (Fig. 5a) can alone or in combination with the WNT/PCP signaling component PRICKLE1 modulate the WNT/β-catenin TOPFlash reporter assay in HEK293T cells. We first found that expression of LRRK2 alone decreased the basal level of TOPFlash activation up to 40% (Fig. 5b, d). This effect was maintained after deletion of the armadillo domain (dHEAT) and lost by deletion of COR domain (KINASE, WD40 and LRRS LRRK2 mutants) (Fig. 5b) pointing out that the RocCOR domains are likely important for WNT/PCP signaling activation and/or WNT/β-catenin inhibition. We also found that PRICKLE1 in combination with LRRK2 induced TOPFlash/β-catenin activation (Fig. 5c). This activation was LRRK2-dependent, as it was reduced by LRRK2 truncations, being the ARMADILLO-ANKYRIN and the LRR domains essential, with a contribution of the WD40 domain (Fig. 5c). LRRK2 most common PD mutations did not inhibit TOPFlash in a significant manner (Fig. 5d). These data suggest that multiple domains of LRRK2 are necessary to regulate WNT/β-catenin signaling and that this regulation is context dependent, since PRICKLE1 is capable of turning LRRK2 from an inhibitor into an activator of this pathway.

### PRICKLE1 reduces the activation of WNT/β-catenin signaling by competing with DVL2 in the presence of LRRK2

It has been previously shown that DVL increases WNT/β-catenin signaling activity [51–53], and that LRRK2 binds to DVL [19], and together they further increase the TOPFlash/β-catenin activity by 2-5 folds depending on the DVL isoform [20]. On the other hand, PRICKLE1 can bind DVL, which is then ubiquitinated and targeted for degradation, leading to the downregulation of WNT/β-catenin signaling [54]. This interaction has been proposed to be a mechanism by which PRICKLE1 regulates asymmetric localization of FZD and DVL across cell-cell contacts from the proximal to the distal side of the cell [55–57]. We therefore examined how LRRK2 and PRICKLE1 modulate the DVL2-induced activation of WNT/β-catenin signaling (Fig. 5e). Our results confirm that DVL2 induces the activation of WNT/β-catenin signaling (~60 fold). This activation was not modified by PRICKLE1, but was increased by LRRK2 (~130 fold compared to control) as previously described [51]. However, the DVL2-LRRK2 dependent activation of WNT/β-catenin signaling was reduced by PRICKLE1 down to ~80 fold. Constitutively active beta-catenin (ca-B-CATENIN) was used as a positive control (~500 fold) to show that the effect of DVL2-LRRK2 was not saturated. These data suggest that the co-localization of LRRK2, PRICKLE1 and DVL2 in signalosome-like structures (Fig. 4c-e) allows PRICKLE1 to inhibit the increase in WNT/β-catenin signaling induced by LRRK2-DVL2.

Combined, our data indicate that the LRRK2-PRICKLE1 protein complex acts as a modulator of WNT/β-catenin signaling, increasing or decreasing its activity in the absence or the presence of DVL2, respectively. This observation is consistent with several studies showing that protein levels, temporal events and the localization of LRRK2 within the cell are important determinants for the function and regulation of LRRK2 activity [20, 58–61].

### Overexpression of LRRK2 in *Xenopus laevis* embryos causes inhibition of WNT/β-catenin signaling and convergence and extension defects

**Fig. 6:**
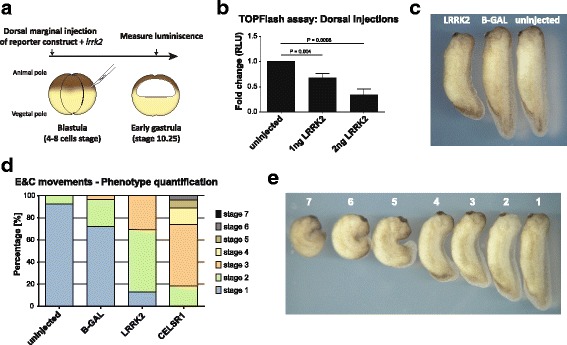
LRRK2 inhibits WNT/β-catenin signaling in vivo and mediates WNT/PCP signaling during *Xenopus* early development. **a**-**b** TOPFlash assay in *X. laevis* embryos. **a** Schematic drawing of the experiment. 1 or 2 ng of *lrrk2* mRNA was co-injected with 200 pg 14XTOPFlash and 25 pg Renilla reporters DNA into dorsal marginal cells at 4 to 8 cell-stage of *Xenopus laevis* embryos. Firefly and Renilla luciferase were measured at stage 10.25. **b** Overexpression of *lrrk2* in *Xenopus laevis* embryos lead to significant reduction of WNT/β-catenin activity in a dose-dependent manner. Two tailed T-test, *N* = 3. **c**-**d** Overexpression of *lrrk2* mediates convergence and extension movement’s defects in *Xenopus* embryos. **c** Typical phenotype of the *lrrk2* overexpressing embryo. 180 pg of *lrrk2* (*left*) or *β-galactosidase* (*right*) DNA was injected in dorsal marginal cells at the 4 to 8-cell stage. **d** Bar chart of convergent extension defects in *lrrk2* (180 pg DNA), *β-galactosidase* (180 pg DNA) and *celsr1* (1 ng mRNA) injected embryos. **e** Categories used for evaluating the convergent extension defects. Grade 1: >0.95; Grade 2: 0.85-0.95; Grade 3: 0.75-0.85; Grade 4: 0.65-0.75; Grade 5: 0.55-0.65; Grade 6: 0.45-0.55; Grade 7: <0.45

We next examined whether LRRK2 in addition of inhibiting WNT/β-catenin signaling in vitro, also does that in vivo. For this purpose we took advantage of *Xenopus laevis,* a well characterized model organism with well-established biological read-outs to examine the activation of WNT signaling pathways [62]. We first co-injected *lrrk2* mRNA (1-2 ng) with the 14XTOPFlash and Renilla reporters’ DNA into dorsal marginal cells at 4-8 cell-stage embryos, and measured luciferase activity at stage 10.25 (Fig. 6a). These experiments revealed that overexpression of *lrrk2* inhibits the WNT/β-catenin pathway in vivo (Fig. 6b), confirming thus our in vitro observations.

Our in vitro findings also provided evidence that LRRK2 inhibits WNT/β-catenin signaling through the activation of the WNT/PCP pathway. It has been shown that the WNT/PCP signaling controls the convergent extension movements [63, 64]. We therefore examined whether *lrrk2* controls these processes during the early development of *Xenopus* embryos. Overexpression of *lrrk2* was performed in the dorsal marginal zone at 4-8 cell stage embryos, and the embryos were subsequently analyzed at stage 32. Interestingly, LRRK2 misexpression resulted in a mild convergent extension defects compared to uninjected or *β-galactosidase* controls (Fig. 6c). In order to perform a more detailed analysis, we used *celsr1* as positive control [26, 65] and evaluated the different grades of the convergent extension defects caused by WNT/PCP pathway deregulation. For that, we measured the length of anteroposterior axis in each embryo, and normalized it by the average length of uninjected embryos. Based on the analysis of numerous embryos (79 uninjected, 29 *β-galactosidase*, 46 *lrrk*2 injection and 27 *celsr1*) (Fig. 6d), we divided the WNT/PCP phenotypes into 7 categories (Fig. 6e). Our results show that 56.5% of *lrrk*2-injected embryos displayed a grade 2 phenotype and 30.4% a grade 3, whereas the phenotype of the uninjected and the *β-galactosidase* group were grade 1 in 92.4% and 72.4% of the cases, respectively. On the other hand, *celsr1,* a strong regulator of WNT/PCP pathway, exhibited more severe phenotypes (grade 2 = 18.5%, grade 3 = 55.6%, grade 4 = 14.8%, grade 5 = 7.4%, grade 6 = 3.7%). Thus, our in vivo data demonstrate that LRRK2 is a novel regulator of the WNT/PCP signaling pathway, as well as an inhibitor of the WNT/β-catenin signaling pathway.

## Discussion

### Identification of novel LRRK2 binding partners: GIPC1 and ILK

Although many LRRK2 substrates have been proposed, the identification of true LRRK2 binding partners at physiological levels and their function remains to be determined. Our IP-coupled MS/MS approach using 2 different in vitro cell types and 2 biochemical methods in combination with our candidate-based approach has provided valuable information about what proteins can bind to LRRK2 after overexpression and at endogenous levels. Here we identify a number of novel interactors that are functionally linked to the WNT/PCP signaling pathway, which have been validated by other biochemical methods. These include GIPC1, ILK, PRICKLE1, CELSR1, FLOTILLIN-2 and CULLIN-3. Interactions of LRRK2 with GIPC1 and ILK were further verified by immunoblotting in vitro and in vivo, in the ventral midbrain of E18.5 mouse embryos.

One of the novel LRRK2 interactors that we identify in our study is GIPC1, an adaptor protein for GPCRs that is involved in vesicle trafficking of multiple receptors [66]. GIPC1 interacts with VANGL2, a key WNT/PCP membrane protein. Disruption of GIPC1 function leads to defects in hair cell maturation and hair follicle orientation, a typical phenotype displayed by WNT/PCP deregulation [21]. Moreover, it has been shown that GIPC1 interacts with dopamine D2 and D3 receptors [67–70] and its mutation results in a reduction of DA neurons leading to locomotion defects in *D. melanogaster* [71]. It is thus conceivable that alterations in the interaction between LRRK2 and GIPC1 may be of importance for the function of mDA neurons, a possibility that remains to be investigated.

There is evidence that LRRK2 binds and phosphorylates focal adhesion kinase (FAK) by which it regulates motility of reactive microglia and its response to brain injury, both of which were impaired in G2019S mutants [72]. The role of LRRK2 in regulating cytoskeletal rearrangements is further supported by our observation that LRRK2 binds ILK, a serine-threonine kinase that remodels the cytoskeleton and the extracellular matrix, leading to changes in cell shape, cell polarity and cell motility [23, 73]. It has been shown that overexpression of ILK activates WNT/β-catenin signaling [38, 39], while down-regulation of ILK leads to planar cell polarity defects and failure in hair-follicle development [23]. Moreover, ILK induces WNT/PCP pathway when it binds to the proline-rich motifs of DVL and subsequently promotes RHOA/CDC42/RAC1-mediated cell motility [22, 74, 75]. This dual activity of ILK towards distinct WNT signaling pathways correlates well with our finding that its binding partner, LRRK2, also modulates the activity of WNT/β-catenin and WNT/PCP signaling depending on the exact composition of the interacting complex and its protein levels. Our findings thus provide a mechanistic basis for how LRRK2 could mediate such effects.

### LRRK2 interacts with two core WNT/PCP signaling components: PRICKLE1 and CELSR1

Using a candidate-based approach we also discovered that LRRK2 specifically interact with PRICKLE1 and CELSR1, two key players of the WNT/PCP signaling pathway. Notably, the localization of LRRK2 in the cell varied depending on its binding partners. CELSR1 and LRRK2 were found at the plasma membrane, corresponding to oligomerized, autophosphorylated LRRK2 which displays high kinase activity [76]. On the other hand, PRICKLE1 and LRRK2 were found in cytoplasmatic puncta, a localization similar to the functional complexes formed by LRRK2 and DVL1/3 [19]. Interestingly, we demonstrate that PRICKLE1-LRRK2 localizes in DVL2 signalosome-like structures and functionally interacts. Indeed, PRICKLE1 turned LRRK2 from an inhibitor to an activator of basal WNT/β-catenin signaling, but it inhibited the DVL2-induced activation of this pathway even in the presence of LRRK2. These findings suggest that not only the localization, but also the functionality of LRRK2, is regulated by its interaction with distinct WNT/β-catenin and WNT/PCP signaling components.

While the reason for the differential localization is unknown, LRRK2 shares several functions with PRICKLE1 and CELSR1. LRRK2 regulates axon-dendrite polarity [77], endocytosis and synaptogenesis [50, 78–81], and neurite branching in human dopaminergic neurons [82]. Similarly, CELSR1 also regulates dendrite and axonal growth in cooperation with FZD, VANGL and DVL [83–85], and PRICKLE1 promotes neurite and axon outgrowth [86]. Moreover, mutations in human PRICKLE1 have been associated to seizures [87], progressive myoclonus epilepsy [88, 89] and autism [90]. These findings suggest that LRRK2, by interacting with CELSR1 or PRICKLE1 may contribute to the regulation of typical WNT/PCP-controlled functions such as synaptogenesis, vesicle trafficking and synaptic plasticity [91–94].

### LRRK2 regulates both WNT/β-catenin and WNT/PCP pathways

It has been previously shown that LRRK2 regulates WNT/β-catenin signaling [19, 20, 95], a pathway that is in most cases negatively regulated by signaling components of the WNT/PCP pathway [25, 42]. LRRK2 has a GTPase activity and binds active RAC1 [96], a small GTPase that (besides other functions) acts downstream of WNT5A-dependent WNT/PCP signaling activation [46, 84, 97]. Interestingly, G2019S and R1441C mutations fail to activate RAC1 [96], and also result in altered levels of WNT/β-catenin signaling [20]. Moreover, a recent study [98] has shown that loss of function of LRRK2 increases WNT/β-catein signaling in cultured fibroblasts and in adult mice in vivo. These mice also exhibit increased bone density and altered tibia shape, a phenotype suggestive of WNT/PCP signaling, which plays a crucial role in limb development [99–101]. Indeed, PRICKLE1 is known to control the growth of long bones and its mutation causes severe skeletal malformations resulting in shorter and wider bones [102]. These data together with our results indicate that alterations in LRRK2 structure and/or function are likely to modify the balance between WNT/β-catenin and WNT/PCP signaling. Strikingly, our results show that LRRK2 has the capacity to either inhibit or upregulate WNT/β-catenin signaling depending on its interacting partners and levels of WNT signaling. Moreover, overexpression of LRRK2 in human cells demonstrated that LRRK2 alone inhibits WNT/β-catenin signaling via its RocCOR domain. This result was confirmed in vivo, in *Xenopus* embryos, where LRRK2 not only inhibited the WNT/β-catenin signaling but also regulated the WNT/PCP signaling pathway, as shown by alteration of convergence-extension movements, the process by which cells intercalate and the embryo elongates. Thus our results provide first evidence for a role of LRRK2 in WNT/PCP signaling pathway.

Core WNT/PCP signaling components such as PRICKLE1 [54] and CELSR1 [27], have been previously found to inhibit WNT/β-catenin signaling. Surprisingly, PRICKLE1 in complex with LRRK2 increased basal WNT/β-catenin signaling, but reduced the DVL-induced activation of WNT/β-catenin signaling. Thus our findings suggest a role of PRICKLE1 and LRRK2 as dual regulators of WNT signaling by switching between WNT/β-catenin and WNT/PCP signaling activity.

## Conclusions

Our study shows for the first time that LRRK2 interacts with WNT/PCP signaling pathway through its physical binding to multiple WNT/PCP regulatory components. By performing a proteomic screening we discovered several candidate LRRK2 interactors, and verified a few proteins, such as GIPC1 and ILK, which bind to endogenous LRRK2 in dopaminergic cells and in mouse ventral midbrain. We also demonstrate that LRRK2 can bind to FLOTILLIN-2, CULLIN-3, CELSR1 and PRICKLE1, which lead to changes in LRRK2 localization. While LRRK2 has been previously shown to activate the WNT/β-catenin pathway, our data suggest that the regulation of WNT signaling is more complex. We found that the formation of specific protein complexes can trigger WNT/PCP signaling and inhibit the WNT/β-catenin pathway both in vitro and in vivo, in *X. laevis*. Our results thus show that LRRK2 works as a dual regulator of WNT/β-catenin and WNT/PCP signaling (as PRICKLE1 and ILK), and suggest a possible additional role of LRRK2 in controlling biological processes regulated by the WNT/PCP pathway and deregulated in PD, such as axon-dendritic polarity and synaptic function.

## Additional files

Additional file 1: Figure S1. Whole immunoblots showing the specificity of the antibodies used in this study. (**A**-**B**) Three different monoclonal anti-LRRK2 antibodies were tested in cells with low levels of LRRK2 CRISPR knockdown (KD) and GFP CRISPR (WT). All three antibodies specifically recognize and pull down LRRK2 in the *substantia nigra* dopaminergic cell line, SN4741. (**C**-**D**) Full size immunoblots show the specificity of the antibodies used in this study. (**D**) Antibodies against different tags were tested on transiently overexpressed tagged proteins in HEK293T cells. The western blot bands corresponded to the correct molecular weight for each protein detected. (EPS 10805 kb)

Additional file 2: Table S1. List of antibodies used in the study with detailed information about the producer and dilutions. (DOCX 17 kb)

Additional file 3: Figure S2. IP-coupled MS/MS revealed a number of proteins involved in different biological processes. (**A**) The silver-stained polyacrylamide gel that was subjected for MS/MS analysis with the strong band corresponding to pulled-down LRRK2 which was overexpressed in HEK293T cells. (**B**) Manual analysis based on Uniprot annotations [34] and published research showed that LRRK2 binding partners have 1) a potential link to WNT signaling, 2) function in the mitochondrial metabolism, 3) regulate the cell cycle, 4) localize into the nucleus, 5) belong among cytoskeletal proteins or 6) have an unknown function. (**C**) The list of LRRK2 interactors that can be potentially involved in WNT signaling. (EPS 4097 kb)

Additional file 4: Table S2. Complete list of identified proteins interacting with either IgG or with LRRK2 in SN4741 cells using two different protocols (“in solution” and “in gel” experiments). The results are searchable with help of various filters set up in the excel file. (XLSX 2501 kb)

Additional file 5: Table S3. Complete list of identified proteins from overexpressed Myc-Lrrk2 experiment using HEK293T. The lists contain either IPMYC sample or IgG sample. The file includes separate runs corresponding to single gel fractions (#1-5, Additional file 3: Figure S2A). Results from all the fractions per each sample are merged in separate sheets and named as IPMyc_merged and IgG_merged. (XLSX 126 kb)

Additional file 6: Table S4. Description of the five most interesting endogenous LRRK2 binding partners extracted from the common hits of IP-coupled MS/MS of endogenous LRRK2 (In-gel vs In-solution) with direct or indirect links to WNT signaling pathways. Basic information about the protein function were drawn on Uniprot [34, 106–123]. (DOCX 64 kb)

Additional file 7: Figure S3. Characterization of the LRRK2 KD SN4741 cell line genomic DNA. (**A**). T7E1 assay shows that the LRRK2 cell line contains monoallelic mutations which is evident by the presence of 3 different bands in gel electrophoresis. Band of 418 bp represents an uncut, wild type sequence of LRRK2. Bands of 254 bp and 162 bp are result of succesful T7E1 cut inside of the mutated sequence. (**B**) The table sums up sequencing results of control GFP SN4741 cell line and LRRK2 KD SN4741 cell line. At the place where sgRNA targetting LRRK2 binds to the genomic DNA and right before the PAM sequence, the LRRK2 KD cell line lost 2 base pairs and gained 3 new base pairs, which caused a frame shift and created a stop codon. (EPS 2148 kb)

Additional file 8: Figure S4. Confirmation of the interaction between LRRK2 and CELSR1 using different conditions. (**A**) Western blotting analysis of co-IP of overexpressed V5-Lrrk2 and Celsr1-EGFP in HEK293T cells. Different vectors were used to confirm LRRK2-CELSR1 interaction. LRRK2 binds to CELSR1 even when pulled-down with different antibody. (**B**) Co-immunoprecipitation of overexpressed LRRK2 with CELSR1 by using different ratio of transfected DNA in HEK293T cells. Efficiency of the LRRK2-CELSR1 pulldown had improved when less CELSR1 and more LRRK2 was transfected. This not only confirms the interaction but also excludes the false positive interactions compared to the negative controls. (EPS 5517 kb)

Additional file 9: Figure S5. Panel of endosomal markers and their relation to LRRK2/PRICKLE1/DVL2 puncta. LRRK2 partialy co-localizes with RAB5A (**A**), and weakly with RAB11, a marker of recycling endosomes (**B**). *Arrows* point at the co-localization. LRRK2 does not co-localize with a marker of late endosomes, RAB7 (*arrowheads*) (**C**). RAB7 strongly co-localizes with PRICKLE1 (**D**). PRICKLE1 and DVL2 co-localize with RAB7 (*arrows*), nevertheless a part of PRICKLE1 is localized outside of the RAB7 positive endosomes (*arrowheads*) (**E**). Nuclear staining by Dapi in blue. *N* ≥ 3. Scale bars show 10 μm. (EPS 94808 kb)

## Abbreviations

BEND3: BEN domain-containing protein 3
CNS: Central nervous system
COR: C-terminal of COR domain
DVL: Segment polarity protein dishevelled homolog DVL
ENA/VASP: Enabled/Vasodilator proteins
GIPC1: PDZ domain-containing protein GIPC1
GPCRs: G protein coupled receptors
ILK: Integrin-linked kinase
LPP: Lipoma-preferred partner
LRP6: Low-density lipoprotein receptor-related protein 6
LRR: Leucine-rich repeats
LRRK2 KD: LRRK2 knock down
LRRK2 WT: LRRK2 wild type
LRRK2: Leucine-rich repeat kinase 2
mDA neurons: mouse dopaminergic neurons
PCP: Planar Cell Polarity
PD: Parkinson’s disease
ROC: Ras of complex protein domain
SDOCH: Sodium deoxycholate buffer
SNpc: *Substantia nigra pars compacta*
VANGL2: Vang-like protein 2
VM: Ventral midbrain
WNT: Wingless/Integration
