# Supplementary material for: A proteomic analysis of LRRK2 binding partners reveals interactions with multiple signaling components of the WNT/PCP pathway
Source: Mol Neurodegener. 2017 Jul 11;12:54. doi: 10.1186/s13024-017-0193-9 (PMC5505151; doi:10.1186/s13024-017-0193-9)
Supplement: Supplementary file 2 — List of antibodies used in the study with detailed information about the producer and dilutions. (DOCX 17 kb) [file 13024_2017_193_MOESM2_ESM.docx]

**Suppl. Table S1:**

|  | **NAME** | **PROVIDER** | **CATALOG NUMBER** | **SPECIES** | **DILUTION** |
| --- | --- | --- | --- | --- | --- |
| **Primary antibodies** | C-MYC | Santa Cruz | sc-40 | mouse | 1:1000 |
|  | C-MYC | Sigma | C3956 | rabbit | 1:2000 |
|  | DVL3 | Santa Cruz | sc-8027 | mouse | 1:500 |
|  | FLAG M2 | Sigma | F1804 | mouse | 1:1000/2000 |
|  | FLAG | Sigma | F7425 | rabbit | 1μg for IP |
|  | GAPDH | Sigma | G8795 | mouse | 1:5000 |
|  | GFP | Abcam | ab-6662 | goat | 1:2000/5000 |
|  | GIPC1 | ProteinTech | 14822-1-AP | rabbit | 1:1000 |
|  | HA | Abcam | ab-9110-100 | rabbit | 1:1000 |
|  | ILK | Origene | TA300878 | rabbit | 1:1000 |
|  | LPP | Novus Biologicals | NBP1-47516 | mouse | 1:1000 |
|  | LRRK2 (MC.028.83.76.242) | Covance/BioLegend | Sig-39840 | mouse | 1:500 |
|  | LRRK2 (MJFF2 (c41-2)) | Abcam | ab-133474 | rabbit | 1:500/1000 |
|  | LRRK2 (UDD3 30(12)) | Abcam | ab-133518 | rabbit | 1:500 |
|  | ROR2 | Sigma | HPA021868 | rabbit | 1:2000 |
|  | V5 | Invitrogene | R960-25 | mouse | 1:1000/2000 |
| **Secondary antibodies** | anti-Mouse-HRP | Sigma | A4416 | goat | 1:5000 |
|  | anti-Rabbit-HRP | Sigma | A6667 | goat | 1:5000 |
|  | anti-Goat-HRP | Sigma | A5420 | rabbit | 1:5000 |
|  | anti-Mouse-Alexa Fluor 488 | ThermoFisher Scientific | A21202 | donkey | 1:1000 |
|  | anti-Mouse-Alexa Fluor 555 | ThermoFisher Scientific | A31570 | donkey | 1:1000 |
|  | anti-Mouse-Alexa Fluor 647 | ThermoFisher Scientific | A31571 | donkey | 1:1000 |
|  | Anti-Rabbit-Alexa Fluor 647 | ThermoFisher Scientific | A31573 | donkey | 1:1000 |
|  | anti-Rabbit-Alexa Fluor 555 | ThermoFisher Scientific | A21206 | donkey | 1:1000 |
|  | anti-Rabbit-Alexa Fluor 488 | ThermoFisher Scientific | A31572 | donkey | 1:1000 |
