# Supplementary material for: A proteomic analysis of LRRK2 binding partners reveals interactions with multiple signaling components of the WNT/PCP pathway
Source: Mol Neurodegener. 2017 Jul 11;12:54. doi: 10.1186/s13024-017-0193-9 (PMC5505151; doi:10.1186/s13024-017-0193-9)
Supplement: Supplementary file 6 — Description of the five most interesting endogenous LRRK2 binding partners extracted from the common hits of IP-coupled MS/MS of endogenous LRRK2 (In-gel vs In-solution) with direct or indirect links to WNT signaling pathways. Basic information about the protein function were drawn on Uniprot [34, 106–123]. (DOCX 64 kb) [file 13024_2017_193_MOESM6_ESM.docx]

**Suppl. Table S4:**

| **ENDOGENOUS LRRK2 CANDIDATES AND CONFIRMED INTERACTORS,**  **AND THEIR LINK TO WNT SIGNALING** | |
| --- | --- |
| *NAME* | *PROTEIN FUNCTION DESCRIPTION, REFERENCES* |
| **GIPC1**  (PDZ domain-containing protein GIPC1) | **Cellular function:** Adaptor protein for G-protein coupled receptors (GPCRs). It is also involved in endocytic trafficking of number of receptors, notably receptor tyrosine kinases. GIPC1 plays a role in cell-surface localization of some transmembrane receptors such as IGF1R, LHCGR and TGFbR3. The PDZ domain of GIPC1 is important for direct interactions with a variety of proteins, including dopamine receptor D2 (DRD2), GLUT1, ENDOGLIN, INTEGRIN A5 and A6, NTRK1 (TRKA), SEMA4C, TGFb RECEPTOR TYPE III (TGFbR3), and VANG-LIKE 2 (VANGL2) [reviewed in [106]. |
|  | **Central nervous system:** Interacts with dopamine D2 and D3 receptors [71-73] through its PDZ domain [70]. A *Drosophila* homolog GIPC is expressed in adult brain, especially in DA neurons and glia, and its loss of function is responsible for locomotor defects and reduced longevity [74]. |
|  | **Link to WNT signaling:** GIPC1 interacts with VANGL2 and in complex with MYOSIN VI regulates its trafficking by removal of VANGL2 from the plasma membrane. Disruption of GIPC1 function leads to hair cells maturation defects such as hair bundle orientation and stronger polarization of VANGL2 in the membrane [21]. Moreover, KERMIT1, a frog ortholog of GIPC1, interacts with WNT receptors FRIZZED-3 and FRIZZLED-7 in *X. laevis* [107]. Drosophila’s Gipc mRNA is upregulated in wing development [108], and its overexpression causes planar cell polarity defects in the wing but not in eye [109]. |
| **LPP**  (Lipoma-preferred partner homolog) | **Cellular function:** LPP is related to the ZYXIN family, and is associated with cell migration, proliferation and transcription. It is overexpressed in cancer patients with leukemia and other types of cancer [110, 111]. |
|  | **Link to WNT signaling:** It is involved in WNT/PCP signaling through its binding to PCP protein SCRIB. This protein complex localizes in cell-cell contacts [112]. LPP and SCRIB cooperate and mediate convergence and extension movements in zebrafish early development. Expression of LPP is decreased in WNT11 morphants and in embryos overexpressing WNT11 [113]. |
| **ILK**  (Integrin-linked protein kinase ) | **Cellular function:** Linked to cell adhesion, cell migration and WNT signaling. It contains ankyrin repeats and serine-threonin protein kinase that directly interact with INTEGRINS B1 and B3. Overexpression of constitutively active ILK leads to a loss of cell-cell-adhesion [76] and tumorigenicity in nude mice [114]. |
|  | **Link to WNT signaling:** Overexpression of ca-ILK causes an upregulation of WNT/Β-CATENIN signaling [42]. WNT-3A treatment upregulates ILK protein expression in vascular smooth muscle cells [115]. It modulates epithelial polarity and matrix formation in hair follicles [23]. ILK binds to DVL and induces WNT/PCP pathway downstream upon BMP2 stimulation in p-AKT-dependent manner [22]. ILK indirectly controls somitogenesis by modulation of GSK3β phosphorylation and downstream activation of Notch signaling [43]. |
| **ZYX**  (Zyxin) | **Cellular function:** Important for formation and regulation of focal adhesion and cell motility but also in transducing signals into the nucleus, cell proliferation and differentiation [34]. |
|  | **Central nervous system:** ZYXIN is a substrate of AMYLOID BETA 1-40 and 1-42 [116] and inhibits SHH signaling during the CNS patterning in *X. laevis* through interaction with the transcription factor GLI1. It has been shown that ZYXIN is crucial for reduction of SHH signaling within the dorsal part of the neural tube of *X. laevis* embryos [117]. ZYXIN1also regulates synapse maintenance in *C. elegans* [118]. |
|  | **Indirect link to WNT signaling:** The LIM domain protein WTIP that is related to ZYXIN family interacts with the receptor tyrosine kinase ROR2 and inhibits canonical WNT signalling [119]. |
| **RAPH1**  (Protein Raph1) | **Cellular function:** Also known as Lamellipodin (LPD). It binds to Enabled/Vasodilator proteins (ENA/VASP), actin-associated regulators of lamellipodial protrusion that co-localize at tips of lamellipodia and filopodia. RAPH1 overexpression accelerates lamellipodial protrusion. Knock-down of RAPH1 causes impairment in lamellipodia formation, and decreases the F-actin content [120, 121]. RAPH1 also mediates F-actin dependent endocytosis of EGFR [122]. |
|  | **Central nervous system:** RAPH1 directly interacts with M-Ras in rat cortical neurons during dendrite development and it is required for M-Ras-mediated dendritic maturation. Knockdown of RAPH1 in cortical neurons reduces dendritic outgrowth and branching. Decreased membrane localization of RAPH1 is responsible for Sema4D-mediated dendrite remodeling [123]. |
